# A Review of Visiting Policies in Intensive Care Units

**DOI:** 10.5539/gjhs.v8n6p267

**Published:** 2015-11-17

**Authors:** Shiva Khaleghparast, Soodabeh Joolaee, Behrooz Ghanbari, Majid Maleki, Hamid Peyrovi, Naser Bahrani

**Affiliations:** 1 Nursing Care Research Center (NCRC), Iran University of Medical Sciences (IUMS), Tehran, Iran; 2 Nursing Care Research Center (NCRC), Nursing and Midwifery School, Iran University of Medical Sciences (IUMS), Tehran, Iran; 3 Mental Health Research Center, Tehran Psychiatric Institute, Iran University of Medical Sciences (IUMS), Tehran, Iran; 4 Department of Echocardiography, Rajaie Cardiovascular Medical and Research Center, Iran University of Medical Sciences (IUMS), Tehran, Iran; 5 Nursing and Midwifery School, Iran University of Medical Sciences (IUMS), Tehran, Iran; 6 Faculty Member, Tehran University of Medical Sciences (TUMS), Tehran, Iran

**Keywords:** intensive care unit, visiting hours, visiting policy

## Abstract

Admission to intensive care units is potentially stressful and usually goes together with disruption in physiological and emotional function of the patient. The role of the families in improving ill patients’ conditions is important. So this study investigates the strategies, potential challenges and also the different dimensions of visiting hours’ policies with a narrative review. The search was carried out in scientific information databases using keywords “visiting policy”, “visiting hours” and “intensive care unit” with no time limitation on accessing the published studies in English or Farsi. Of a total of 42 articles, 22 conformed to our study objectives from 1997 to 2013. The trajectory of current research shows that visiting in intensive care units has, since their inception in the 1960s, always considered the nurses’ perspectives, patients’ preferences and physiological responses, and the outlook for families. However, little research has been carried out and most of that originates from the United States, Europe and since 2010, a few from Iran. It seems that the need to use the research findings and emerging theories and practices is necessary to discover and challenge the beliefs and views of nurses about family-oriented care and visiting in intensive care units.

## 1. Introduction

### 1.1 Background

Admission to intensive care units (ICUs) is potentially stressful and usually goes together with disruption in physiological and emotional function of the patient (Vandijck et al., 2010). The critical condition of these patients is a source of pain and stress and even crisis for themselves and their families. Thus paying attention to specific needs of the patients and their families is one the essential principles of responsiveness by the doctors and nurses working in intensive care units. The role of the families in improving ill patients’ conditions is important and the health service team works in a patient and family oriented system. Findings of many studies in this context show that the “open visiting” strategy is an essential need of the patients and their families. Clinical instructions in many countries recommend an open visiting policy for the family oriented care of the intensive care unit (Spreen & Schuurmans, 2011). A flexible and open visiting policy can have a positive effect of the patients’ condition and eventually their families and help them cope with this crisis and be more satisfied (Haghbin, Tayebi, Abbasian, & Haghbin, 2011; Whitton & Pittiglio, 2011).

However, open visiting hours can cause some major concerns for the nurses and the doctors: 1) it can increase the patients’ physiological stress; 2) it can interfere with nurses’ care and 3) it can lead to physical and emotional exhaustion of the families (Berwick & Kotagal, 2004). Evidence based studies show that the visiting strategy for patients in an intensive care units must be based on the patients’ needs and have no limitations regarding time, duration and/or number of visitors at the bedside (Spreen & Schuurmans, 2011).

### 1.2 Aim

Traditionally, intensive care unit visiting hours in Iran follow constrained and strict rules. Despite the importance of this issue in our country, there have been few studies concerning this issue and we believe that in order to implement the best visiting hour strategy, a transition in the culture is needed. This should be based on a review of texts relating to visiting hours strategies, this study investigates the strategies, potential challenges and also the different dimensions of visiting hours’ policies.

## 2. Methods

### 2.1 Study Design

A narrative review about visiting policies in Intensive Care Units was performed. Narrative reviews are the traditional approach mainly based on the experience and subjectivity of the author (Cipriani & Geddes, 2003).

### 2.2 Search Strategy

The search was carried out in scientific information databases such as Web of science, PubMed, CINAHL, Google Scholar and All EBM Reviews (OVID) using keywords “visiting policy”, “visiting hours” and “intensive care unit” with no time limitation on accessing the published studies in English. In order to access published Iranian research, some Iranian databases, including the Iran Medex, Google, Scientific Information Database (SID) and the published information database of the country (Magiran) were searched with the same keywords in Farsi, the Persian language.

### 2.3 Inclusion and Exclusion Criteria

We included all reviews, analytical designs and descriptive studies reported on visiting policies and visiting hours published in peer-review journals. Only articles in English or Persian were included. Studies not related to ICUs were excluded too.

### 2.4 Article Categorization

**Figure 1:**
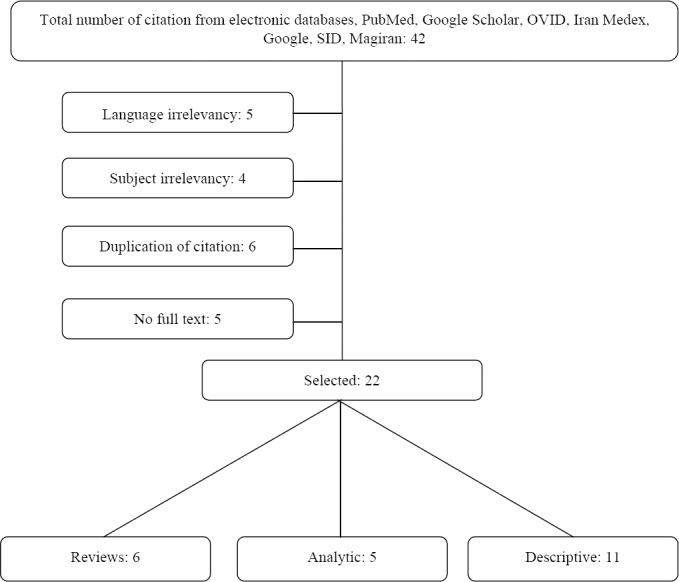
Study selection flow chart

From 42 total articles gathered in our search, 22 were selected for reading. Figure 1 presents a summary of the study selection process.

## 3. Results

### 3.1 Review Studies

A total of 42 articles were found with from 1997 to 2013 with the keywords. Of these, 22 articles conformed to our study objectives of addressing strategies, potential challenges and different dimensions of visiting strategies in ICUs. The remainder did not particularly investigate visiting hours in intensive care units and were therefore outside the scope of this research. Some abstracts were also investigated when the full text was not accessible. Some instructions in the United States, UK, India, Belgium, Italy, and France and hospital statements related to visiting hours strategies were also studied. As seen in Figure 1, 22 studies were reviewed, including six reviews, five analytic and descriptive studies, and six were review studies.

### 3.2 Studies in Iran

From 22 studies investigated, only English and three Persian articles in the internal scientific information databases, particularly discussed visiting hours in ICUs in Iran (Table 2).

**Table 1:** An overview of studies conducted about visitation hours in intensive care unit

Summary of the articles	Title	Authors (year)
Noordermeer et al. (2012)	Visiting Policies in the Adult Intensive Care Units in the Netherlands: Survey among ICU Directors	The objective was to evaluate visitation policies in the adult intensive care units in the Netherlands. A questionnaire on visitation policy was completed by nurses in all intensive care units in the Netherlands. Most ICUs (87.1%) of intensive care units had limited visitation policies and their main reason was preventing interference with the work program of the care staff and the privacy of the patients. This study showed that the open visiting policy leads to better results for reduced stress for patients and families.

Whitton et al. (2011)	Critical care open visiting hours	In order to understand and raise awareness about the effects of open visitation hours for patients, family members, and nurses of intensive care units, ten0 experimental studies were reviewed. Most of them discussed the urgent need of intensive care unit patients. The studies showed that an open visiting policy improves quality of care to the satisfaction of patients, family members, and nurses.

Davidson et al. (2007)	Clinical practice guidelines for support of the family in the patient-centered intensive care unit: American College of Critical Care Medicine Task Force 2004-2005	In order to develop clinical practice guidelines for the protection of patients and families in ICUs, members of the US Department of Intensive Care reviewed more than 300 related studies published between 1980 and 2003; most of them showed the benefits of an open visitation policy.

Cook (2006)	Open visiting: Does his benefit… (Dissertation)	This review study examines the visiting hours, procedures, patient preference, physiological effects, and advantages and disadvantages of open visiting and states that nurses are an important factor in determining the visiting policy.

Bray et al. (2004)	British Association of Critical Care Nurses position statement on the use of restraint in adult critical care units	The critical condition of the patients in ICUs or the possibility of death in this unit makes it necessary for their families to visit them. Visiting policies have created several challenges in this regard. Proponents of limited visit policies argue that open visits increase noise, compromise patient safety, limit the space, and waste nurses’ time.

Clarke et al. (2001)	The needs of children visiting on adult intensive care units: a review of the literature and recommendations for practice	The objective was to review available research and studies to determine nursing interventions for the need of children to visit adult parents in ICUs. The review showed that the need of adult patients in intensive care units to have the visits of their children is not adequately considered, and more studies should be conducted to evaluate the short- and long-term effects on children during visits to their very sick family members or friends.

**Table 2:** Studies on the visiting hours in intensive care units in Iran

Summary of the articles	Title	Study design	Authors (year)
Haghbin et al. (2013)	Descriptive cross-sectional	Visiting hour policies in intensive care units, southern Iran	The present study sought to evaluate the visiting hours and policies in intensive care units in Fars province (southern Iran); 71 intensive care units were examined. The results showed the same pattern of limited ICU visiting hours. The authors suggested that policy change is an urgent need and should consider the cultural aspects and the rights of patients by health care staff.
Rahmani et al. (2013)	Semi-experimental	The effect of planed meetings on the physiologic indicators of the patients who suffer from Acute Coronary Syndrome	The study was carried out to determine the effect of planned visits on the physiological parameters of patients with acute coronary syndrome. The results showed that the supporting effect of planned visits was higher than limited visits, and therefore it reduced the physiological indices and thereby helped to improve the quality of treatment.
Salavati et al. (2012)	Semi-experimental	The effects of planed meeting on the physiologic indicators of the patients admitted to intensive care units	The study examined the effects of planned visits on cardiac indices of patients in ICU. The results indicated that planned visits for the presence of family and friends at bedside did not cause clinically significant changes for the worse in cardiovascular parameters. Therefore there was no reason for the restriction of visits to patients in ICUs.
Ghiyasvandian et al. (2010)	Semi-experimental	The effect of open visiting on intensive care nurses’ beliefs	The objective was to analyze the effect of change in open visiting policies on nurses’ views about nursing care. Nurses’ views were negative before the change in the policy. Distribution of scores of nurses’ views before and after changing the visiting policy showed a significant difference. The prevailing factor in nurses’ views was a social factor, especially the role of the colleagues and supervisors, as well as management and policies.

## 4. Discussion

Traditionally intensive care units have been open and welcoming and this may be a reason for patients’ concern about their privacy. The critical conditions of these patients or the possibility of their death make the need for their family to visit crucial. This has become a challenge because of the physical space constraints. Some studies indicate that visits should be possible at any time and are necessary, while others state that preserving the patients’ privacy and the dignity of their visitors is imperative. The visiting methods and policies have caused many arguments regarding how visiting in ICUs should be managed (Bray et al., 2004). No major model has yet been approved for handling the visiting strategies (Carlson, Riegel, & Thomason, 1998; Olsen, Dysvik, & Hansen, 2009; P Ramsey, Cathelyn, Gugliotta, & Glenn, 1999).

### 4.1 Visiting Hours

‘Visiting’ takes on a wide spectrum of meanings in the hospital environment. Limited visiting is a method that allows visitors presence during a specific time, while open visiting allow visitors to be with their family and friends at any time they want. The human conditions and compassion call for “flexible” visits between these two extreme strategies. Other names such as contractual, structured or unlimited visiting have also been used to describe what is meant. These strategies are different for public or private hospitals, special emotional conditions of the patients and various cultural differences (Cook, 2006).

#### 4.1.1 Limited Visits

Haghbin et al. (2011) believe that this method is traditionally more beneficial to the hospital and the care staff that the patients and their families need. However, many ICUs are still using limited visiting. This method is related to the traditional beliefs and that increasing the rest or sleeping time of patients is important and gives nurses more control, preventing crowds in any one room (Haghbin et al., 2011), prevents visitors tampering with reports and avoids the presence of rude or provocative visitors. Studies show that most intensive care units in UK follow this strategy. However in the United States a more open and flexible strategy is observed. A strategy of limiting the age and number of visitors are allowed but can be asked to leave the patient in special cases, disregarding any requests by the patient or the family (Cook, 2006; Spreen & Schuurmans, 2011).

Vandijck et al. have shown in their studies that most ICUs in western Europe still follow a restricted policy in which the time and duration is priori specified (Vandijck et al., 2010). Evidence show that ICU strategies in 70% percent of the hospitals in United States limit family visits (Bell, 2011) because some ICU nurses believe that family visits cause more physiological stress for patients, interfering with nursing care, cause emotional exhaustion patients and their families, endanger patient safety if emergency access to the patient is needed, and increase the possibility of infection (Kirchhoff & Dahl, 2006; Roland, Russell, Richards, & Sullivan, 2001; Smith, Medves, Harrison, Tranmer, & Waytuck, 2009). However, some evidence does not support these beliefs (Berwick & Kotagal, 2004; Bray et al., 2004; Fumagalli et al., 2006).

#### 4.1.2 Open Visits

This strategy allows families to visit their patient at any time they want during the day (Bray et al., 2004; Spreen & Schuurmans, 2011). Evidence shows that the presence of family and their help with ill patients’ care is useful (Haghbin et al., 2011). Being together in time of crisis and stress is respectful of the rights of patients and their families. However, it seems that nurses regularly agree to limit visits to enforce power and control, while yet being responsible for effective communication with the families, given that ICUs are not limited to the physical and physiological needs of patients (Cook, 2006). Several studies show that the presence of family and friends increases the satisfaction of patients and their family due to promoting the guarantee of patient care (Haghbin et al., 2011). This is especially significant when patients are intubated and cannot speak. The presence of visitors can improve the personnel’s communication, understanding, care and satisfaction (Davidson et al., 2007; Soumagne et al., 2011; Whitton & Pittiglio, 2011). Reviews (Roland et al., 2001), descriptive (Gonzalez, Carroll, Elliott, Fitzgerald, & Vallent, 2004) and intervention (P Ramsey et al., 1999; Priscilla Ramsey, Cathelyn, Gugliotta, & GLENN, 2000) studies show that open visiting strategies are preferred by patients and improve the satisfaction of their visitors and mitigate their stress. A few research studies have compared the amount of nurses’ and patient visitors’ satisfaction in different ICUs’ visiting strategies (P Ramsey et al., 1999) and visiting hours and their potential challenges in Iran. In 2008, Ghiasvandian et al. have shown that the nurses were skeptical before changing the visiting strategy from limited to open; however, they were positive after changing the strategy. According to the findings of that study the predominant factor in the nurses’ beliefs was influenced by the role of their colleagues, supervisors, policy makers and management (Ghiyasvandian, Abbaszadeh, Ghojazadeh, & Sheikhalipour, 2009). It is clear that the rules and policies on visiting change from a country to another and depend on the culture, the space available in the hospital and the ICU, the geographical location, the accessibility of facilities and technologies, the personnel’s willingness to accept future changes, evolutions and different routines (Haghbin et al., 2011).

Despite the fact that the information based on evidence shows that the visiting strategy in ICUs must be according to the patients’ needs and should not have limits regarding time, duration or the number of visitors. The clinical instructions should recommend strategies for unlimited visiting in ICUs because of the family oriented care. Visiting rules in ICUs are usually set by the ICU staff. They must balance the needs of the members of the family, the needs of the patients to rest and the needs of the nurses to provide patients care. Being with the patients in ICU is as important for their family as being informed about their illness. Olsen et al. (2009) have reported that the patients and their families preferred different visiting hours, from half an hour a day to 1-2 hours a day or 35-55 minutes for 3-4 times a day (Olsen et al., 2009).

### 4.2 Patients Preferences

Several studies have shown that patients prefer flexible visiting strategies. Open visiting hours realize the families’ needs and have a positive impact on the patients, improve patient satisfaction and may even reduce the stay in hospital (Davidson et al., 2007; Noordermeer, Rijpstra, Newhall, Pelle, & van der Meer, 2013; Soumagne et al., 2011; Whitton & Pittiglio, 2011). Gonzalez et al. (2004) have shown that visiting is a non-stressful experience for visitors. They help to interpret the information given by health care providers, and they provide information that is helpful to nurses to understand the patients’ personality and coping style. Patient preferences are different depending on age, illness-related characteristics, personality, type of the unit, sustainable or unsustainable hemodynamic state, being intubated and the differences between needs of men or women. Researches show that although patients prefer open visiting hours, sometimes they desire limited visiting, especially when patients do not have good feelings about their family or the dynamism of the family is not welcomed. In that case, instead of applying an overall scheme and strategy to visit, some personal limitations can be made before the visitors’ arrival (Cook, 2006).

### 4.3 Physiological Impacts

Traditionally, some nurses believe that an open visiting strategy is potentially harmful to ill patients. They believe that it increases the intracranial pressure, blood pressure, heart rate and causes premature atrial and ventricular contractions. There is no research available to support negative physiological impacts of family visits on patients. To the contrary, researchers suggest that open visiting reduces stress and leads to tranquility and thus helps patients to rest. Different studies have shown that not only has there been no increase in cardiovascular indicators (e.g. blood pressure, heart rate, etc.) in patients having visitors but in fact was even considerably lower (Berwick & Kotagal, 2004) and had no destructive physiological effect on the patients (Berwick & Kotagal, 2004; Cook, 2006; Fumagalli et al., 2006).

Salavati et al. (2012) have also shown that in Iran scheduled visiting hours for family and friends to patients does not have any significant clinical impact on cardiovascular indicators. Thus, according to that study, there is no reason to limit the visits for patients in ICUs. Rahmani et al. (2013) indicated that the supportive impact on patients of scheduled visits is higher than limited visiting and that this reduces the cardiovascular indicators, helping to improve the quality of treatment in acute coronary syndrome (ACS) patients in ICUs.

### 4.4 Interfering With the Patient Care Schedule

Another reason for disagreement on unlimited (open) visiting hours is that it interferes with nurses’ and doctors’ work and makes patient care more difficult for them. Studies show that families create more opportunities to facilitate patient-staff communications and even train patients to communicate better with staff (Berwick & Kotagal, 2004). Members of the family also provide more effective feedback to patients than nurses and doctors, which lead to better working conditions. Family members can be politely asked to temporarily leave the room in urgent cases or for specific procedures (Garrouste-Orgeas et al., 2008; Noordermeer et al., 2013).

### 4.5 Family or Friend’s Exhaustion

Some researchers believe that open visits create problems for families or friends as they come with their own needs. However, the fact is that open visiting hours help to relieve the anxiety of the families as they can spend more time with their patients and feel more secure in being with them than not being there. Berwick et al. have shown that open visiting hours has a positive effect on 88% of the families and reduces anxiety in 65% of them (Berwick & Kotagal, 2004).

### 4.6 Benefits of Open Visiting Hours

The foundation of patient care is the needs and demands of the patient. Patients having visitors are more satisfied as this is part of realizing the needs of their family and of their own. Several studies show that not only has visiting no undesired physiological effect on the patients, but provides them with comfort and convenience, reduces cardiovascular complications, creates a feeling of security and satisfaction for the patients and the family and improves their communication with the staff. The presence of the family is in most cases effective; e.g. providing information about patient history, helping nurses choose physical care, motivating the patient and helping in improvement and positive reinforcement of the patient (Garrouste-Orgeas et al., 2008; Rahmani, Motahedian Tabrizi, & Rahimi, 2013; Spreen & Schuurmans, 2011). Although in recent decades an open visiting strategy has been used extensively in special care baby and children’s units, his is still not the case for adult ICUs and this is a contradiction in health care systems. Thus in order to provide holistic care, patients should be considered as a member of a family unit (Anzoletti, Buja, Bortolusso, & Zampieron, 2008; Clarke & Harrison, 2001; Spreen & Schuurmans, 2011).

### 4.7 Drawbacks of Open Visits

Despite nurses understanding the importance of open visiting, implementing this strategy still faces many obstacles in case of the ICUs (Cook, 2006). The lack of human resources, rapid expansion of technology and severe patient illness status makes the family’s presence a reason for nurses feeling threatened or disappointed. The constant stream of visitors’ questions, the effort to control nurses, requesting case reviews and reports, tending to choose particular nurses for their patient’s care, insisting on precise information before accepting even the slightest changes in treatment and delaying the time of medication administration are all reasons given for not having open visiting hours. The safety and privacy of intensive care management may also be threatened by open visiting. I despite all this, evidence show that approximating and managing the risks and problems of open visiting is generally possible (Berwick & Kotagal, 2004).

### 4.8 Nurses as an Effective Factor in Visiting

Nurses have a primary role in visiting hour’s strategies (Simon, Phillips, Badalamenti, Ohlert, & Krumberger, 1997). Despite evidence that the presence and intervention by family members is useful in patient care, some head nurses still prefer to limit family visits and use power to control the visitors (Simon et al., 1997). Their rationale is often based on experience and the judgment by nurses instead of substantial research. Apparently nurses believe that visitors cause stress, exhaustion and negative physiological effects in the patients, yet these assumptions are not based on research. In fact, research has shown the positive effects of family visits and its comforting effect on physiological responses by patients (Berwick & Kotagal, 2004; Cook, 2006; Rahmani et al., 2013; Salavati, Najafvandzadeh, Oshvandi, Homayounfar, & Soltanian, 2013). Anzoletti et al. (2008) believe that what must be considered about visiting ill patients is to accept the best outcomes for patients and this is realized by considering the needs of the patients and their families. In summary, the nurses’ opinions drive visiting strategies in all ICUs, and thus any efforts by management to change the strategy without accepting or changing the nurses’ views and believes is hardly possible. As the nurses’ traditional reasons for supporting limited visits strategy are not validated, using research evidence and worldwide consumer rights as more important, nurses should also accept this change of perspective and pay attention to this matter. These perspectives can be changed with constant professional trainings (Anzoletti et al., 2008).

## 5. Conclusion

The trajectory of current research shows that visiting in ICUs has, since their inception in the 1960s, always considered the nurses’ perspectives, patients’ preferences and physiological responses, and the outlook for families. However, little research has been carried out and most of that originates from the United States, Europe and some from New Zealand and Australia (Cook, 2006) and since 2010, a few from Iran. It seems that the need to use the research findings and emerging theories and practices is necessary to discover and challenge the beliefs and views of nurses about family-oriented care and visiting in ICUs. This requires time to publish research findings so that nurses can choose the best decision concerning visiting strategies and consider the best interests for patients. Constant evaluation and training also help nurses to choose the best visiting strategy. Hospital managers, physicians, social services and support groups are other helpful sources in this context.

Nurses must be aware of the significance of open visiting for patients’ and their families’ benefits and consequently visitors must be aware of the patients’ privacy, current geographical space for care provision and their ability to help in patient care. Individual differences between patients and families (age, illness related issues, culture, personality, etc.) and allowing nurses to control and organize the ICU in developing the visiting strategy must be considered to result in an appropriate agreement between patients, families and staff concerns. Not only does open visiting not harm patients, but it creates a support system for them and forms familial environments (Spreen & Schuurmans, 2011). Open visiting hours reinforce the trust in the families concerned and result in better working communications between hospital staff and family members. Hospitals that want to use open visiting hours in their ICUs must first implement such a strategy for some months and ask the patients, families, nurses and doctors to monitor the transition effects openly and objectively. The result will certainly be better for patients and families who are the center of care (Berwick & Kotagal, 2004). There is a need for more research in this context in health care systems in Iran. The concept of family-oriented and comprehensive care needs to be defined and developed in the ICUs in Iran. More investigations concerning different cultural groups are also crucial. Visiting strategies and their effects on patients’ sleeps and rest, stress, pain level, bed rest duration, complications and the number of days being ventilated is also important to study. Comparative research of perceptions and experiences of the nurses and families regarding visiting in ICUs is a research priority for future studies.
